# Are Clinicians Contributing to Excess African American COVID-19 Deaths? Unbeknownst to Them, They May Be

**DOI:** 10.1089/heq.2020.0015

**Published:** 2020-04-17

**Authors:** Adam J. Milam, Debra Furr-Holden, Jennifer Edwards-Johnson, Birgete Webb, John W. Patton, Nnayereugo C. Ezekwemba, Lekiesha Porter, TomMario Davis, Marius Chukwurah, Antonio J. Webb, Kevin Simon, Geden Franck, Joshua Anthony, Gerald Onuoha, Italo M. Brown, James T. Carson, Brent C. Stephens

**Affiliations:** ^1^ Michigan State University College of Human Medicine, Flint, Michigan, USA.; ^2^ Johns Hopkins University, Bloomberg School of Public Health, Baltimore, MD, USA.; ^3^ Wayne State University School of Medicine, Detroit, Michigan, USA.; ^4^ Stanford University School of Medicine, Stanford, California, USA.; ^5^ University of Texas Southwestern Medical Center, Dallas, Texas, USA.; ^6^ Duke University School of Medicine, Durham, North Carolina, USA.; ^7^ Texas Back Institute, Plano, Texas, USA.; ^8^ Boston Children's Hospital, Boston, Massachusetts, USA.; ^9^ Memorial Hermann Health System, Houston, Texas, USA.; ^10^ Meharry Medical College, Nashville, Tennessee, USA.; ^11^ Lone Star Orthopedic Institute, Kingwood, Texas, USA.; ^12^ Health First, Melbourne, Florida, USA.

**Keywords:** implicit bias, African American, health inequities

## Abstract

African Americans are overrepresented among reported coronavirus disease 2019 (COVID-19) cases and deaths. There are a multitude of factors that may explain the African American disparity in COVID-19 outcomes, including higher rates of comorbidities. While individual-level factors predictably contribute to disparate COVID-19 outcomes, systematic and structural factors have not yet been reported. It stands to reason that implicit biases may fuel the racial disparity in COVID-19 outcomes. To address this racial disparity, we must apply a health equity lens and disaggregate data explicitly for African Americans, as well as other populations at risk for biased treatment in the health-care system.

African Americans are overrepresented among reported coronavirus disease 2019 (COVID-19) deaths in the United States. In Michigan, for example, where African Americans represent 14% of the population, they account for more than 30% of COVID-19 cases and more than 40% of deaths.^1^ The disparity in Chicago is more troubling. African Americans represent 70% of the COVID-19 deaths but only 29% of the city's population.^1^ There are a multitude of factors that may explain the African American disparity in COVID-19 deaths, including higher rates of comorbid health conditions such as hypertension and cardiovascular disease, barriers to health-care access, and differences in cultural attitudes.^2^ While these individual-level factors predictably contribute to disparate COVID-19 outcomes, systematic and structural factors have not yet been reported.

It is conceivable, even plausible, that African Americans are dying from COVID-19 at higher rates because these patients are more likely to have a “do not resuscitate” directive, commonly referred to as a DNR. The extant literature demonstrates African Americans, compared to whites, are far less likely to endorse a DNR and, in fact, have a preference for life-prolonging care.^3–5^ For example, Mack et al. examined differences in end-of-life care among a sample of patients with advanced cancer. They found that African Americans were nearly three times less likely than whites to have DNR orders.^3^ Medical mistrust and fear of receiving inadequate medical care are consistently hypothesized mediators for fewer DNR orders among African American patients.^3–5^ During the COVID-19 pandemic, however, it is possible that despite patients' (and their families') wishes, medical personnel are making the decision of whether to apply life-prolonging measures. It is also possible that African American patients are disproportionately being encouraged to agree to a DNR.

The lack of published data or statistical reports on DNR rates for African Americans with COVID-19 leaves many questions unanswered. Are African Americans being steered toward DNRs during the COVID-19 pandemic? Are African Americans less likely to be offered early intubation and ventilation during COVID-19? Are physicians and other medical personnel less aggressive with treatment or disproportionately not offering African American patients all treatment options?

This premise—that a patient would be encouraged to change their code status to DNR or would be otherwise denied lifesaving treatment because of their race—has precedence. Multiple studies support this supposition. Ezer et al. found that blacks had lower rates of lung resection (the definitive treatment for early-stage lung cancer), even after controlling for access to care, pre-existing health conditions, and other demographics, including sex and socioeconomic status.^6^ The study investigators inferred that the underlying bias of the surgeon who consulted for the lung resection may have contributed to racial differences in the rates of lung resection. Stated differently, the bias of the surgeon may have negatively impacted the course of treatment and subsequent treatment outcomes for African Americans. Another study found that blacks diagnosed with bone fractures were less likely to be prescribed opioids for pain control compared to white patients.^7^ This study also found that black patients who *were* prescribed opioids were less likely to receive a concurrent prescription for naloxone, a commonly used reversal agent to prevent and reduce opioid overdose death. We surmise that implicit bias is the culprit.

Implicit (or unconscious) biases are the stereotypes or preferences for or against groups of people that are held by individuals who are unaware of them and, as a result, are unable (or highly unlikely) to change.^8^ This is distinct from the conscious thoughts and feelings that individuals hold about groups of people. Unconscious bias is most problematic because people are unaware that they have a preference for or against people based on factors such as race, age, or gender—to name but a few. A systematic review of studies assessing racial/ethnic implicit biases using implicit association tests among health-care providers was published in 2018.^8^ Three major themes related to health-care providers' implicit biases and patient interactions emerged: (1) the majority of health-care providers demonstrated implicit biases against African Americans; (2) African American health-care providers demonstrated lower implicit biases overall; and (3) stronger implicit biases among providers were associated with worse patient–provider communication.^8^ These biases often persist, even after accounting for patient-level factors.

It stands to reason that implicit biases are impacting conversations that well-trained and well-meaning medical personnel are having with African American COVID-19 patients and their families about code status and disease management. This alone will likely result in a smaller number of critically ill African American patients with COVID-19 being placed on a ventilator—a virtual death sentence. Admittedly, the health-care system was not prepared to handle the large influx of patients during the COVID-19 pandemic. Overburdened providers are now forced to make tough decisions with scarce resources, and their unconscious biases will likely amplify the health disparities gap.

The problem of implicit bias in the health-care system is not new. In fact, it was noted in the 2003 Institute of Medicine Report, *Unequal Treatment*. The COVID-19 pandemic should be a call for radical intervention and policies to ensure that providers are screened for implicit bias and provided evidence-based feedback and training to move those biases from the unconscious to the conscious.^2^ There is evidence that once people are aware of their biases and receive training and feedback, they *can* change their behaviors.

Moving forward, we must apply a health equity lens and disaggregate data explicitly for African Americans, as well as other populations at risk for biased treatment in the health-care system, including women and non-gender confirming sexual minorities, incarcerated and other institutionalized persons, the disabled, the elderly, non-native English speakers, undocumented residents—the list is endless. The health-care system and all of its agents, policy makers, and elected officials are all accountable to ensure that our social and political determinants of health no longer disproportionately burden the groups most at risk for unfair or inequitable treatment but strive to deliver our systems best care. The bridge between public health and medicine also needs to be strengthened such that the findings of population studies can be more readily translated to improve our current health-care delivery system.

*Or*… maybe we are okay with the alarming rate of COVID-19 deaths among African Americans, applying our usual explanations that they are sicker, poorer, and have less access to care. Maybe health disparities are the inalienable truth and *status quo* in America. Accepting this reality will not increase the capacity of well-meaning medical staff to help all people fairly and belies the self-evident principles on which this country was founded. We (a collective of African American physicians and public health professionals) are sounding the alarm that we seize this opportunity to address the health disparities and systematic inequities that continue to result in premature mortality and shortened life expectancy among African Americans and other disadvantaged, disenfranchised, and already marginalized populations.
